# Intracranial Large Artery Abnormalities and Association With Cerebral Small Vessel Disease in CADASIL

**DOI:** 10.3389/fneur.2020.00726

**Published:** 2020-08-18

**Authors:** Chen Zhang, Wei Li, ShaoWu Li, SongTao Niu, XinGao Wang, Xueying Yu, ZaiQiang Zhang

**Affiliations:** ^1^Department of Neurology, China National Clinical Research Center for Neurological Diseases, Beijing Tiantan Hospital, Capital Medical University, Beijing, China; ^2^Department of Neuroimaging, Beijing Neurosurgical Institute, Beijing Tiantan Hospital, Capital Medical University, Beijing, China

**Keywords:** CADASIL, *NOTCH3*, cerebral small vessel disease, intracranial large artery, WMH

## Abstract

**Background and objective:** Cerebral autosomal dominant arteriopathy with subcortical infarcts and leukoencephalopathy (CADASIL) is an inherited systemic arteriopathy, the classic feature of which is small vessel lesions. Studies on intracranial large arteries in CADASIL are not common. We aim to evaluate intracranial large arteries, describing the characteristics of large arteries in CADASIL and their association with cerebral small vessel associated lesions.

**Methods:** Consecutive CADASIL patients from a single-center prospective cohort were analyzed. Brain magnetic resonance imaging and magnetic resonance angiography were performed to assess the intracranial large arteries and cerebral small vessels associated lesions' neuroimaging.

**Results:** The study included 37 CADASIL patients. Of the patients, 28 of them (75.7%) had intracranial large artery abnormalities. Eighteen (48.6%) had congenital variations such as fenestration, vertebral artery (VA) hypoplasia and agenesis, or common trunk and fetus posterior cerebral artery. Seventeen (45.9%) had acquired anomalies such as arterial stenosis, prolongation, or tortuosity (seven of them had both congenital and acquired anomalies). CADASIL patients with anterior circulation middle cerebral artery (MCA) or internal cerebral artery (ICA) severe stenosis were more likely to have ipsilateral asymmetric white matter hyper-density (WMH) distribution. Patients with posterior circulation VA hypoplasia had a higher prevalence of posterior subcortical zone dominant WMH distribution.

**Conclusion:** CADASIL patients can demonstrate various intracranial large artery abnormalities which might influence the development of microangiopathy. Assessment of great vessels seems essential in CADASIL.

## Introduction

Cerebral autosomal dominant arteriopathy with subcortical infarcts and leukoencephalopathy (CADASIL) is an inherited systemic arteriopathy caused by mutations of the *NOTCH3* gene (1). The arteriopathy is characterized by thickening of the arterial wall, deposits of granular osmiophilic material (GOM), and degeneration of smooth-muscle cells (SMCs) (2). Central nervous system disorders and cerebral small vessel associated neuroimaging abnormalities are common characteristics of CADASIL. However, studies on the characteristics of intracranial large vessels in CADASIL are rare. Whether great vessels play a role in the pathophysiology of cerebral small vessel disease (SVD), and the relationship between great vessels and small vessels in CADASIL, remains unclear. In this study, we describe the intracranial large artery abnormalities and the association of cerebral large artery and SVD-related imaging in a series of CADASIL patients.

## Methods

### Study Population

We analyzed the data from a series of 46 consecutive patients admitted to Beijing Tiantan Hospital with a genetically or pathologically confirmed diagnosis of CADASIL between 2014 and 2018. The genetic or pathological screening was triggered in suspected cases with marked leukoencephalopathy on neuroimaging and at least one of the following: young age of stroke onset, migraine with aura, cognitive decline, gait disturbance, and/or family history of ischemic stroke or cognitive dysfunction. Only those who were over 18 years old were included. Nine patients without magnetic resonance angiography (MRA) were excluded, which left 37 patients in the case series. We also recruited 38 controls in patients with sporadic small vessel disease, matched for age and gender.

### Clinical Data

Collected data included demographics (age, sex) and vascular risk factors (hypertension, smoking, hyperlipidemia, and diabetes mellitus). Hypertension was defined as previous diagnosis of high blood pressure (>140/90 mmHg) or use of antihypertensive treatment. Smoking was recorded as present in the case of current or previous history. Hyperlipidemia and diabetes were defined as previous diagnoses or current use of antihyperlipidemic and antidiabetic drugs, respectively.

### Standard Protocol Approvals, Registrations, and Patient Consent

This study was performed with approval from and in accordance with the guidelines of the Ethics Committee of Beijing Tiantan Hospital, and informed consent was obtained from all patients or their families.

### Neuroimaging Acquisition and Analysis

Images were obtained using a 3.0T magnetic resonance imaging (MRI) scanner (Magnetom Trio Tim; Siemens) and included whole brain T2-weighted images (repetition time [TR]/echo time [TE] 4,500/94 ms, 5 mm slice thickness, 1.5 mm interslice gap), fluid-attenuated inversion recovery (FLAIR; TR/TE 9,000/81 ms, inversion time [TI] 2,500 ms, 5 mm slice thickness, 1.5 mm interslice gap), sagittal T1-weighted images (TR/TE 2300/2.3 ms, 1 mm slice thickness, 0 mm interslice gap), and susceptibility-weighted imaging (SWI); repetition time [TR]/echo time [TE] 29/20 ms, 1.2 mm slice thickness, 0 mm interslice gap). Magnetic resonance angiography (MRA) was performed using the 3D TOF technique (TR 22 ms, TE 3.5 ms), 600 um slice thickness.

MRA was performed to assess intracranial large arteries including the internal carotid artery (ICA), middle cerebral artery (MCA), anterior cerebral artery (ACA), vertebral artery (VA), basilar artery (BSA), and posterior cerebral artery (PCA). VA hypoplasia was defined as a diameter ≤ 2 mm (3) involving the unilateral or bilateral artery, or an artery thinner than the other side. BSA elongation was defined as the height of bifurcation above the dorsum sellae (4); BSA tortuosity is assessed using a laterality score (0–3 score, higher score indicates more severe tortuosity) (4). We used a dichotomized classification of normality (0 score) and tortuosity (1–3 score).

Axial T2 and FLAIR were performed for WMH detection. We applied Fazekas Scale to assess the severity of WMH (5). MRIcro was used for computer-assisted determination of WMH volume (6, 7). Periventricular (PV) white matter hyperintensity (WMH), which touches the ventricles, was analyzed (8). We defined and visually assessed three different WMH patterns on FLAIR MRI (**Figure 2**). These included a symmetric pattern-WMH that was distributed symmetrically, a left-right asymmetric pattern-WMH that was located predominantly in one side, and an anterior-posterior asymmetric pattern-WMH that was predominantly in contact with the posterior bilateral ventricular horns (the posterior WMH volume was larger than the anterior WMH).

Enlarged perivascular spaces (EVPS) were assessed in the basal ganglia and centrum semiovale on axial T2-weighted imaging, using a 4-point visual rating scale (0 = no EPVS, 1 = < 10 EPVS, 2 = 11–20 EPVS, 3 = 21–40 EPVS, and 4 = >40 EPVS) (8–11). We used a dichotomized classification of EPVS in accordance with previous studies (8, 11, 12). Total SVD score [An ordinal “SVD score” (range 0–4)] which combines individual MRI feature (WMH, CMBs, PVS and lacunes) of SVD to capture the “total SVD burden” was assessed (13).

All MRI analyses for the presence, site, number, and size of the lesions (WHM, lacuna, perivascular space, and microbleeds) were assessed in line with the Standards for Reporting Vascular Changes on Neuroimaging (STRIVE) recommendations (14) by two investigators independently-blinded to clinical and genetic information. The concordance was studied and any divergence was reviewed by a third expert neuroradiologist. The kappa value of interrater agreement was 0.79 for the left-right asymmetric pattern (*p* < 0.01) and 0.81 for the anterior-posterior asymmetric pattern (*p* < 0.01).

### Statistics

The statistical analysis was conducted using SPSS version 22.0 software. Continuous variables were compared using *t*-test or the Mann-Whitney *U*-test. Categorical variables were compared using Fisher's exact test. Demographic, clinical, and neuroimaging characteristics of CADASIL with cerebral large artery stenosis and those without stenosis were compared, using 2-sample *t*-test, Mann-Whitney *U*-test, and Fisher's exact test as appropriate. The frequency of WMH distribution in CADASIL patients with cerebral large artery involvement was compared by a Fisher's exact test. A two-sided *p* < 0.05 was considered to be statistically significant.

## Results

### Baseline Characteristics and Frequency of Abnormal Intracranial Large Artery in CADASIL

**Figure 1:**
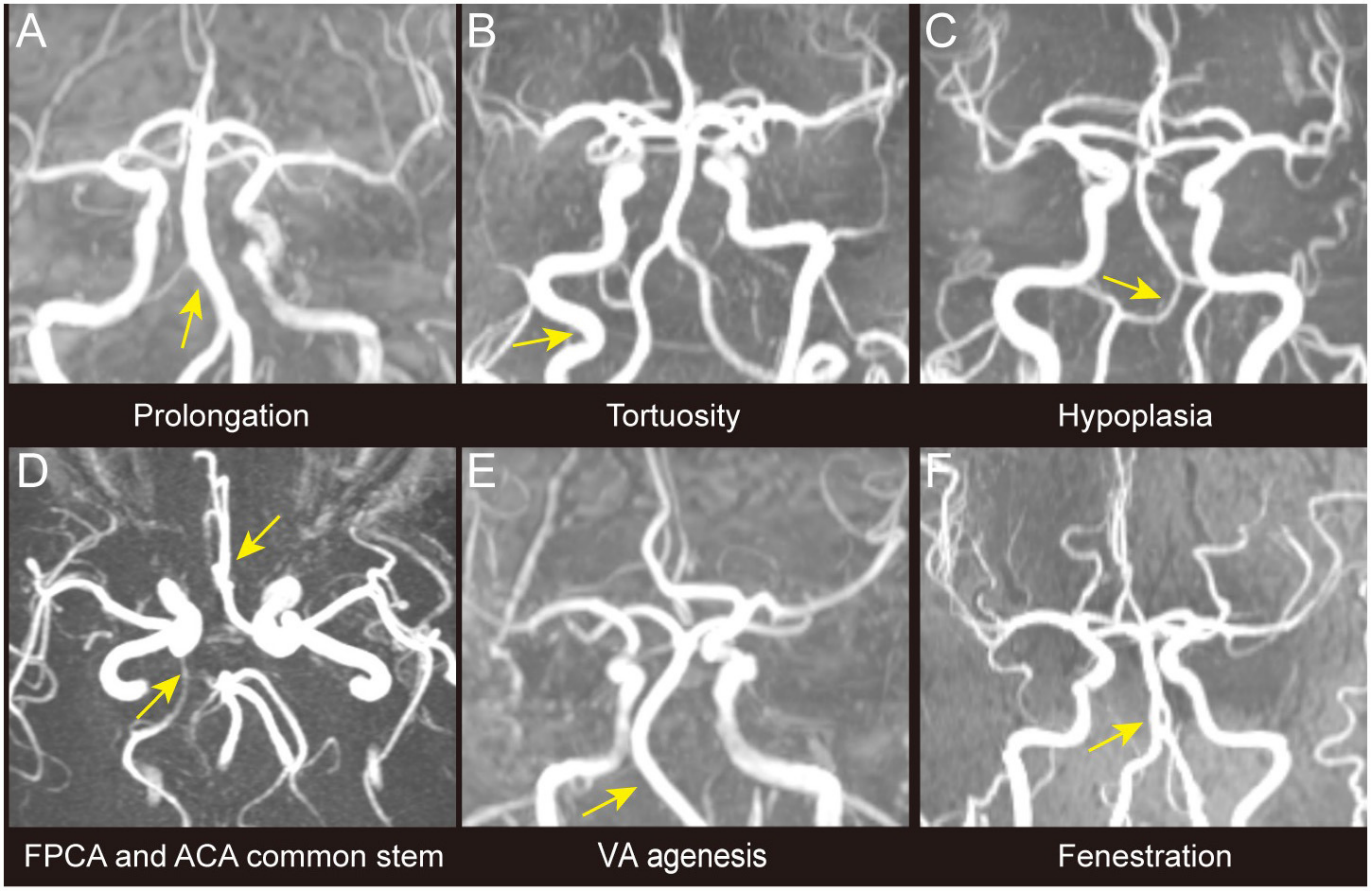
Different types of intracranial large artery abnormalities of CADASIL. The figure shows different congenital variations including the congenital variations (VA hypoplasia, ACA shares common trunk, FPCA, VA agenesis, and basilar artery fenestration) and acquired anomalies (artery prolongation and tortuosity) observed among patients with CADASIL in our study. Only stenosis is not demonstrated in this figure. **(A)** BA prolongation. **(B)** Internal carotid artery tortuosity. **(C)** VA hypoplasia. **(D)** ACA shares common trunk and FPCA. **(E)** VA agenesis. **(F)** BA fenestration. ACA, Anterior cerebral artery; BA, Basilar artery; FPCA, Fetal posterior cerebral artery; VA, Vertebral artery.

**Figure 2:**
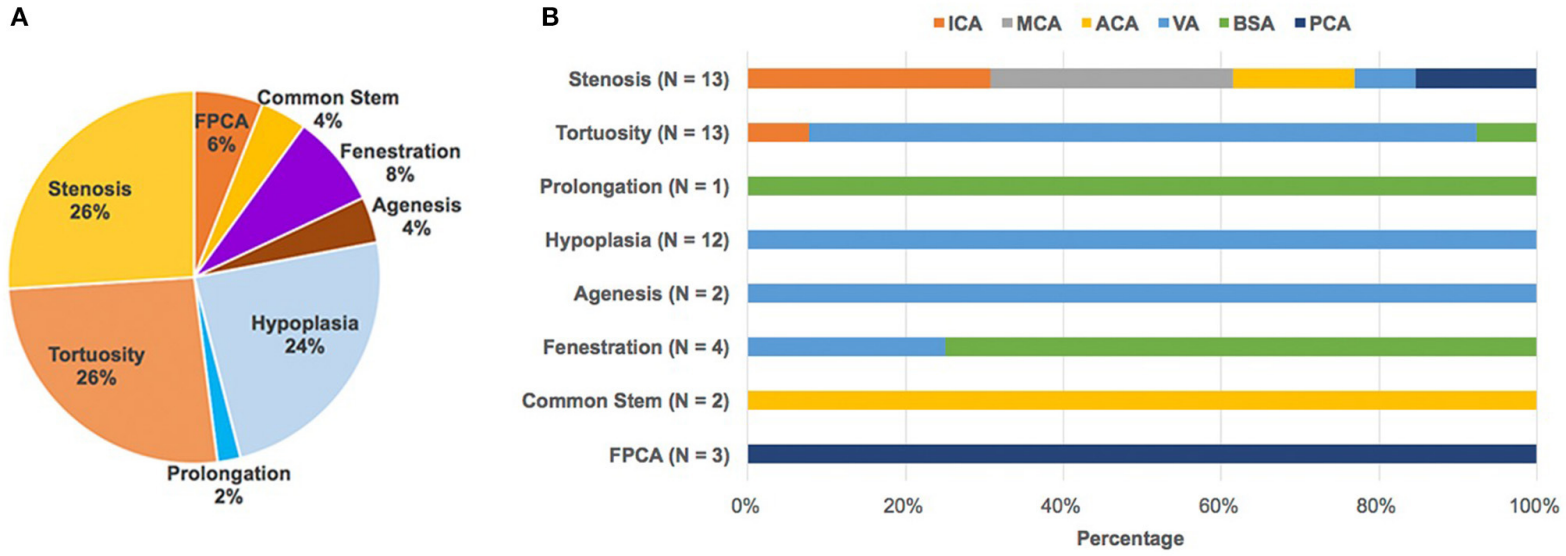
Abnormal intracranial large artery findings and its spectrum of CADASIL. **(A)** In total, 50 intracranial large artery abnormalities including stenosis (*n* = 13), tortuosity (*n* = 13), prolongation (*n* = 1), hypoplasia (*n* = 12), agenesis (*n* = 2), fenestration (*n* = 4), common stem (*n* = 2), and FPCA (*n* = 3) were detected in 28 patients, with 16 patients (43.2%) harboring more than one type of intracranial large artery abnormality. **(B)** Shows the distribution of the involved artery. The number was counted according to the involved artery. ACA, Anterior cerebral artery; BA, Basilar artery; FPCA, Fetal posterior cerebral artery; ICA, internal carotid artery; MCA, middle cerebral artery; PCA, fetal posterior cerebral artery; VA, vertebral artery.

Characteristics of the study population are presented in Table 1. The study included 37 CADASIL patients in total. There were 17 males and 20 females aged 47.5 ± 9.7. A total of 28 of the 37 patients in the study (75.7%) had intracranial large artery abnormalities (Table 1, Figure 1). Eighteen (48.6%) patients had congenital variations and 17 (45.9%) patients had acquired anomalies (seven of whom had both congenital and acquired anomalies). The congenital variations consisted of basilar artery (BSA) fenestration [*n* = 4 of 37, (10.8%)], vertebral artery (VA) hypoplasia [*n* = 12 of 37, (32.4%)], VA agenesis [*n* = 2 of 37, (5.4%)], anterior cerebral artery (ACA) shares common trunk [*n* = 2 of 37, (5.4%)], and fetal posterior cerebral artery [FPCA, *n* = 3 of 37, (8.1%)]. The acquired anomalies included artery stenosis [*n* = 8 of 37, (21.6%)], tortuosity [*n* = 13 of 37, (35.1%)], and prolongation [*n* = 1 of 37, (2.7%)] (Table 1). Sixteen (43.2%) patients harbored more than one type of intracranial large artery abnormality; the distribution of the involved artery was shown in Figure 2. Among the CADASIL patients with cerebral arterial stenosis (*n* = 8), the anterior circulation was involved in five; the posterior circulation in one and two had both anterior and posterior circulation affected. Only one of them was symptomatic. Two patients had severe arterial stenosis; the other six patients had mild stenosis. In the control group, 19 of 38 (50.0%) patients had intracranial large artery abnormalities; only FPCA or ACA shares common trunk had significant difference (13.5% vs. 0.0, *p* = 0.025, Table 2). We can also see that control patients had a much higher rate of risk factors for SVD with statistically significant differences regarding high blood pressure, smoking, and diabetes (Table 2).

**Table 1:** Principal characteristics of the CADASIL patients in this study.

**Characteristics**	**Total CADASIL (*n* = 37)**
**Risk factors**	
Age at time of study entry, *y*, (mean ±*SE*)	47.5 ± 9.7
Male, *n* (%)	17 (45.9)
Hypertension, *n* (%)	10 (27.0)
Smoking, *n* (%)	10 (27.0)
Hypercholesterolemia, *n* (%)	15 (40.5)
Diabetes, *n* (%)	2 (5.4)
**Clinical and genetic characteristics**	
Disease duration, year, (median, range)	6.5 ± 5.5
Ischemic stroke, *n* (%)	26 (70.3)
Hemorrhagic stroke, *n* (%)	5 (13.5)
Cognitive impairment, *n* (%)	30 (81.1)
**Large arteries neuroimaging characteristics**	
Artery stenosis, *n* (%)	8 (21.6)
Tortuosity, *n* (%)	13 (35.1)
Prolongation, *n* (%)	1 (2.7)
BSA fenestration	4 (10.8)
VA hypoplasia or agenesis	14 (37.8)
FPCA and ACA share common trunk	5 (13.5)
**Small vessels neuroimaging characteristics**	
Fazekas score (IQR)	3 (2–3)
Presence of lacuna, *n* (%)	24 (64.9)
Presence of CMB, *n* (%)	19 (51.4)
Presence of EPVS, *n* (%)	31 (83.8)

*ACA, anterior cerebral artery; BSA, basilar artery; CADASIL, cerebral autosomal dominant with subcortical infarcts and leukoencephalopathy; CMB, cerebral microbleeds; EPVS, enlarged perivascular spaces; FPCA, fetal posterior cerebral artery; IQR, interquartile range; SE, standard error; SVD, small vessel disease; VA, vertebral artery*.

**Table 2:** Comparison of demographic and neuroimaging characteristics between CADASIL and leukoaraiosis without genetic SVD.

**Characteristics**	**CADASIL (*n* = 37)**	**Control (*n* = 38)**	***P*-value**
**Risk factors**			
Age at time of study entry, *y*, (mean ±*SE*)	47.5 ± 9.7	51.3 ± 7.7	0.066
Male, *n* (%)	17 (45.9)	20 (52.6)	0.647
Hypertension, *n* (%)	10 (27.0)	31 (81.6)	0.000
Smoking, *n* (%)	10 (27.0)	21 (55.3)	0.019
Hyperlipidemia, *n* (%)	15 (40.5)	12 (31.6)	0.476
Diabetes, *n* (%)	2 (5.4)	8 (21.1)	0.086
**Large vessels neuroimaging characteristics**			
Artery stenosis, *n* (%)	8 (21.6)	4 (10.5)	0.222
Tortuosity, *n* (%)	13 (35.1)	8 (21.1)	0.206
Prolongation, *n* (%)	1 (2.7)	0 (0.0)	0.493
BSA fenestration	4 (10.8)	1 (2.6)	0.200
VA agenesis, *n* (%)	2 (5.4)	3 (7.9)	1.000
VA hypoplasia	12 (32.4)	6 (15.8)	0.111
FPCA or ACA share common trunk	5 (13.5)	0 (0.0)	0.025
**WMH characteristics**			
Fazekas score (IQR)	3 (2-3)	2 (2-3)	0.158

*ACA, anterior cerebral artery; BSA, basilar artery; CADASIL, cerebral autosomal dominant with subcortical infarcts and leukoencephalopathy; FPCA, fetal posterior cerebral artery; IQR, interquartile range; SE, standard error; SVD, small vessel disease. VA, vertebral artery*.

### Comparison of Abnormal and Normal Intracranial Large Arterial in CADASIL

We also compared in the CASADIL group patients with and without abnormal intracranial large arteries. The mean age of patients with abnormal intracranial large arteries was 46.6 ± 10.7, compared to 50.3 ± 5.6 for patients with normal MRA (*p* = 0.190). There were no significant differences in sex, cerebral vascular risk factors, clinical characteristics, and small vessel related imaging characteristics. The comparison of patients with intracranial large artery stenosis (*n* = 8) and without (*n* = 29) were compared; we found a higher percentage of male patients in the group with stenosis (7/8, 87.5%) than in the group without stenosis (10/29, 34.5%, *p* = 0.014). Patients with stenosis also had a higher presence of lacunes (*p* = 0.032), and a higher number of lacunes (*p* = 0.036), compared to the group without senosis (Table 3).

**Table 3:** Comparison of demographic, clinical, and neuroimaging characteristics between intracranial large artery stenosis and non-stenosis CADASIL patients.

**Characteristics**	**Intracranial large artery stenosis (*N* = 8)**	**Intracranial large artery non-stenosis (*N* = 29)**	***P*-value**
Age at time of entry, year, (mean ±*SE*)	45.9 ± 3.2	48.0 ± 1.9	0.592
Male, *n* (%)	7 (87.5%)	10 (34.5%)	0.014
Hypertension, *n* (%)	2 (25.0%)	8 (27.6%)	1.000
Diabetes, *n* (%)	0 (0.0%)	2 (6.9%)	1.000
Hyperlipidemia, *n* (%)	1 (14.3%)	0 (0.0%)	0.233
Smoking, *n* (%)	4 (50.0%)	6 (20.7%)	0.174
Disease duration, year, (median, range)	5 (0.3–16.0)	5 (0.7–23)	0.868
Ischemic stroke, *n* (%)	7 (87.5%)	19 (65.5%)	0.391
Number of attacks, (median, range)	2 (0–5)	2 (0–5)	0.793
mRS, (median, range)	3 (1–5)	1 (0–5)	0.104
Fazekas score, median (IQR)	3 (2–3)	2 (2–3)	0.207
Total WMH, ml, median (IQR)	98.5 (54.3–138.8)	70.5 (37.6–130.4)	0.327
Presence of lacune, *n* (%)	8 (100%)	16 (55.2%)	0.032
No. of lacuna, median (IQR)	3 (2–12)	2 (0–5)	0.036
Presence of CMB, *n* (%)	4 (66.7%)	15 (57.7%)	1.000
No. of CMB, median (IQR)	4 (0–30)	1 (0–22)	0.724
Presence of EPVS, *n* (%)	8 (100%)	23 (74.2%)	0.566
CSO-EPVS (>20), *n* (%)	3 (37.5%)	13 (44.8%)	1.000
BG-EPVS (>20), *n* (%)	3 (37.5%)	21 (72.4 %)	0.100
Atrophy, *n* (%)	5 (62.5%)	16 (43.2%)	0.254
Total SVD score, (median, range)	4 (3–4)	5 (1–4)	0.067

*BG, basal ganglia; CSO, centrum semiovale; CADASIL, cerebral autosomal dominant with subcortical infarcts and leukoencephalopathy; CMB, cerebral microbleeds; EPVS, enlarged perivascular spaces; IQR, interquartile range; No, number of; SE, standard error; SVD, small vessel disease; WMH, white matter hyperintensity*.

### WMH Distribution in CADASIL With Abnormal Intracranial Large Artery

**Figure 3:**
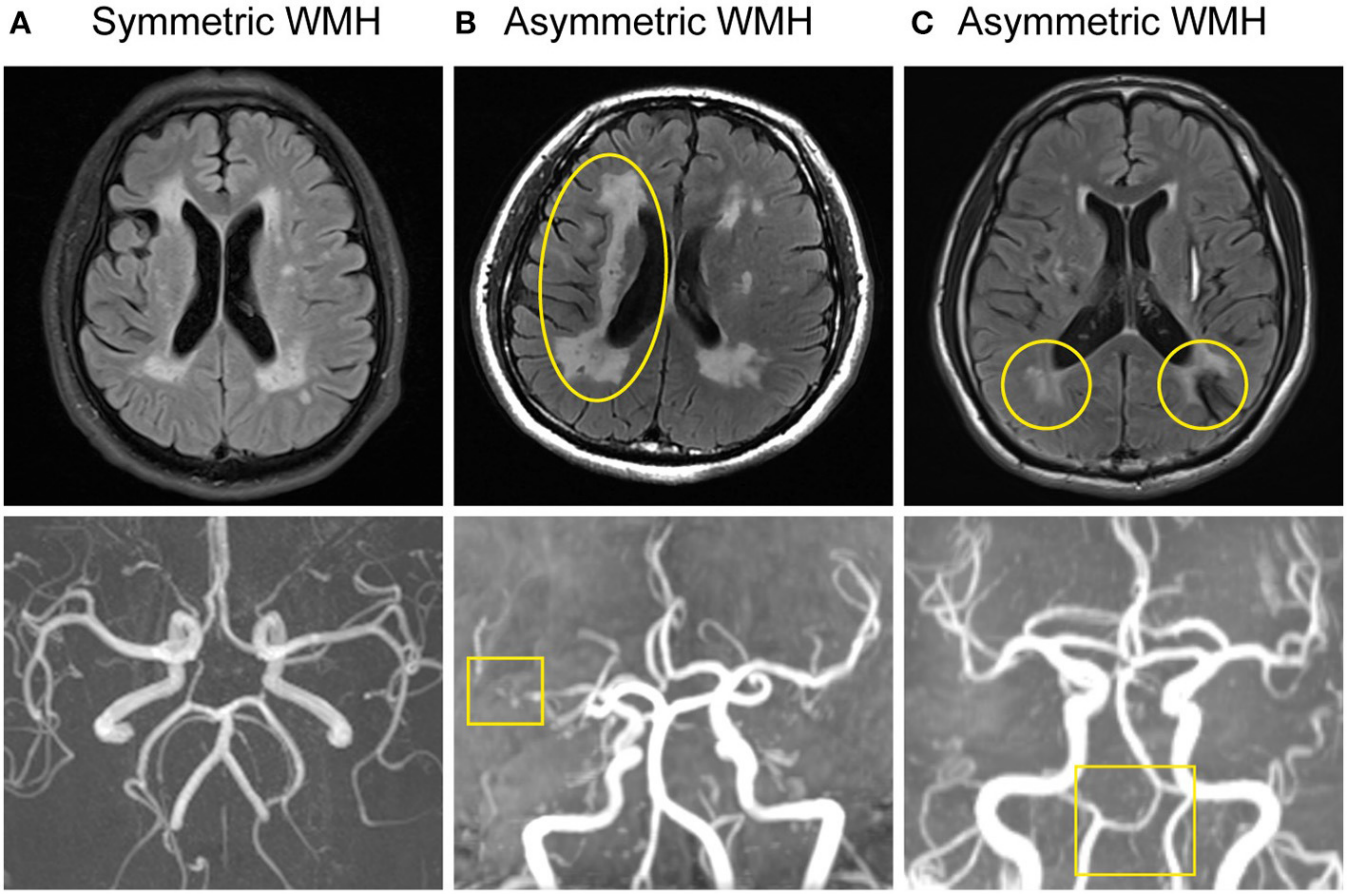
Large artery disorders and WMH distribution of CADASIL. **(A)** Normal intracranial large artery and symmetric WMH distribution. **(B)** MRA showed right MCA horizontal segment severe stenosis. Asymmetric WMH was seen on FLAIR, predominantly located in the right side, presenting with unilateral dominant WMH pattern (left-right asymmetric pattern). **(C)** MRA demonstrated VA hypoplasia. FLAIR displayed asymmetric WMH distribution, mainly in the posterior horn of the bilateral ventricle, presenting with a posterior WMH pattern (anterior-posterior asymmetric pattern).

**Figure 4:**
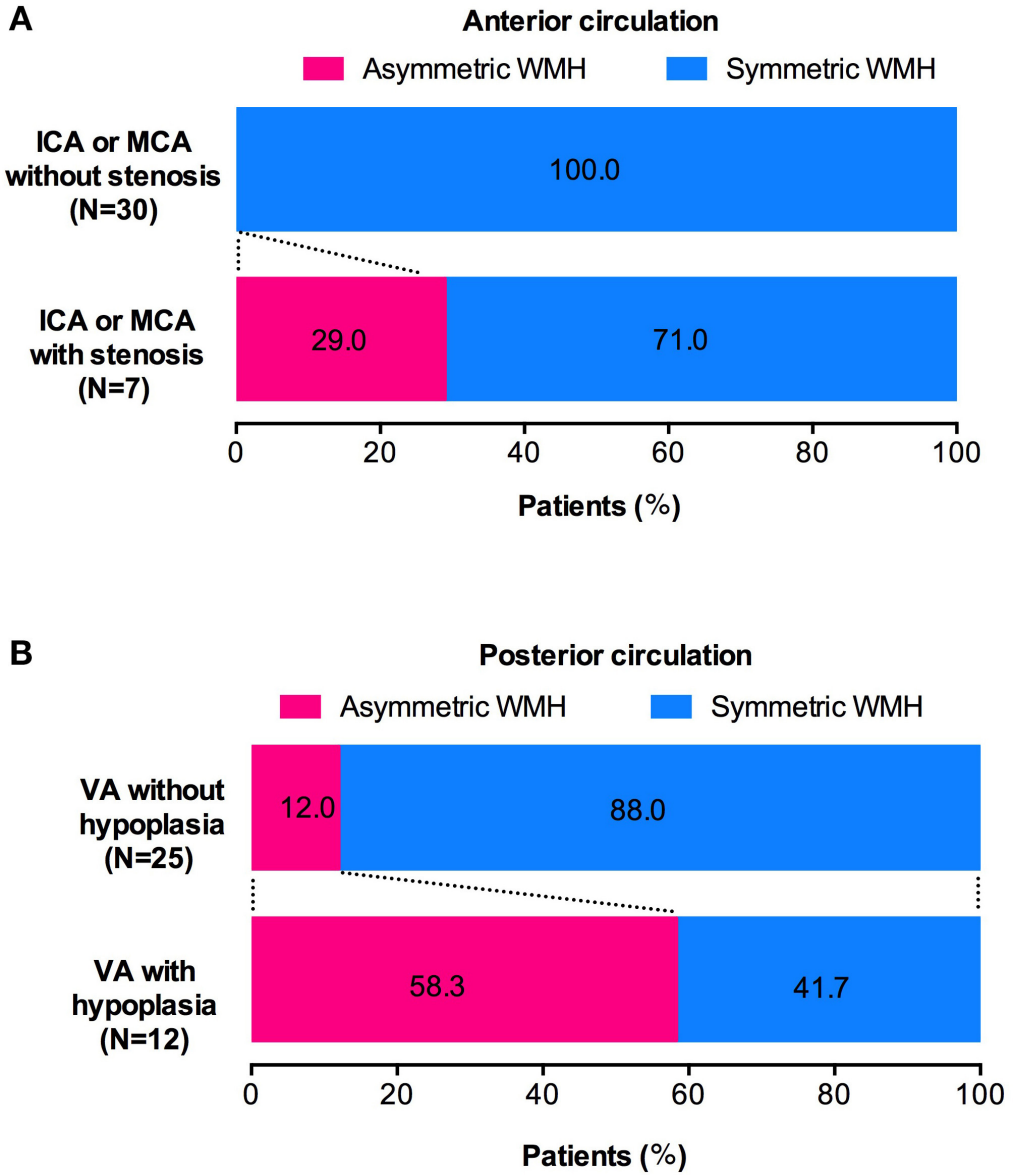
Association of intracranial large artery abnormality and WMH distribution. **(A)** CADASIL patients with unilateral ICA or M1 segment of MCA stenosis have a higher prevalence of ipsilateral dominant WMH pattern (WMH left-right asymmetry). **(B)** CADASIL patients with VA hypoplasia are more likely to have posterior zone dominant WMH pattern (WMH anterior-posterior asymmetry).

The WMH distribution between patients with intracranial large artery stenosis of anterior circulation and those without stenosis showed that patients with ICA or M1 segment of MCA stenosis were more likely to have unilateral asymmetric WMH distribution [Figures 3, 4, *p* = 0.032, OR = 27.73, CI (1.17–660.10)], and the degree of stenosis was ≥50%. The WMH distribution between patients with vertebral artery hypoplasia and those without was compared; patients with VA hypoplasia had a higher prevalence of posterior periventricular WMH distribution [Figures 3, 4, *p* = 0.006, OR = 10.27, CI (1.52–15.58)].

## Discussion

Our preliminary results suggest that patients with CADASIL may demonstrate various intracranial large artery abnormalities, including congenital variations (basilar artery fenestration, VA hypoplasia or agenesis, ACA shares common trunk, and FPCA) and acquired anomalies (stenosis, tortuosity, and prolongation). The group with intracranial large artery stenosis had a higher rate of lacunes at MRI. In addition, CADASIL patients present with an asymmetric WMH pattern may be associated with corresponding intracranial large artery disorders.

Intracranial arterial variants are frequent findings in the general population (15). Although a majority of the variations have little significant clinical impact, some may predispose individuals to the development of aneurysms or ischemic stroke (15, 16). Arterial fenestration has been identified in 10.5% of angiographic studies (17), the frequency of basilar artery fenestration was 2.5% (17) and about 5% in a post-mortem study (18). In our study, the frequency of arterial fenestration was 10.8% and all the locations were basilar arteries; although, this high rate among patients with CASADIL did not reach statistical significance when compared with control (*p* = 0.200). The reason for the relatively higher percentage of basilar artery fenestration may be attributed to the different study population and the ethnicity. Jong-Ho Park et al. (3) reported the incidence of hypoplastic vertebral artery to be 26.5% in a normal group and 35.2% in patients with ischemic stroke; our prevalence (27%) was consistent with that of the normal population. A previous study showed the frequency of vertebral artery agenesis was 3.4% (19) in healthy patients and 4.6% in asymptomatic people (20), similar to the 5.4% found in the current study. The prevalence of the absence of A1 segment (ACA shares common trunk) was reported to be 1–2% in a microsurgical anatomical study (21) and 9% (22) in a retrospective study with patients who underwent carotid and/or cerebral computed tomography angiography (CTA) examinations, and the present observations (5.4%) are compatible with published data. Kovac et al. found the FPCA in 23.3% of a prospectively reviewed study involving patients performing CTA examinations (15). In the present study, the FPCA was detected in 8.1% of the CADASIL patients, which was lower than the previously observed prevalence; this discrepancy might be attributable to the different study population and the ethnicity.

Interestingly, we found a relationship between intracranial large artery abnormality and SVD in CADASIL with a possible influence from the large artery disorders on the distribution of WMH. Generally, the WMH distribution is symmetric in CADASIL (2). Nonetheless, some cases in this study showed asymmetric WMH patterns, which can be divided into two types: left-right and anterior-posterior asymmetric distributions. The unilateral dominant WMH pattern was located in the anterior circulation artery supply area, which is associated with MCA or ICA severe stenosis. The posterior bilateral ventricular dominant WMH pattern was located mostly in the posterior circulation supply area, which is accompanied with VA hypoplasia. These special neuroimaging spatial distribution patterns may be an additional marker of CADASIL patients with intracranial large artery abnormalities.

Although CADASIL with small vessel diseases has been reported worldwide, the features of intracranial large artery involvement is rare. Only a few studies described the prevalence of large artery involvement in CADASIL, and most of them focused on the report of stenosis. A study from Korea revealed large cerebral artery stenosis or agenesis in 12 out of 49 (24.5%) CADASIL patients (23). Another Korean study found that five out 13 (38%) had angiographic abnormalities: three had MCA stenosis, one had distal vertebral artery stenosis, and one had proximal internal carotid artery stenosis and vertebral artery hypoplasia (24). The prevalence of large artery stenosis in our study is eight out of 37 (21.6%), which is similar to the above studies. More studies are warranted to verify the high incidence of large artery involvement (75.7%) observed in our study, and to elucidate the pathology features of the abnormal vessels.

Histologic studies of CADASIL have shown deposition of GOM in the basement membrane of SMCs and degeneration of SMCs (2), which results in the loss of the vasomotor reactivity of small vessel, leading to impaired autoregulation and cerebral hypo-perfusion (24–26). The total blood flow and cerebrovascular reactivity are inversely associated with WMH (26–29). The large artery abnormality (e.g., severe stenosis) may change the hemodynamics and may exacerbate the ipsilateral WMH. Furthermore, one study from Finland demonstrated increased thickness of the wall of white matter arterioles and the presence of stenosis of vascular lumen (30). In addition, the combination of *NOTH3* genotype, environmental factors, traditional cerebral vascular risk factors, and ethnicity might contribute to the complexity of CADASIL phenotype.

Our work identifies the characteristics of various intracranial large artery abnormalities, and shows that there is an association between the intracranial large artery and SVD-related imaging markers: patients presenting with an asymmetric WMH pattern may correlate with corresponding intracranial large artery disorders. Intracranial large artery evaluation is necessary in CADASIL but is not confined to small vessels. Due to the small sample size, our findings will need to be further studied, to better understand the mechanism of the vascular pathology and determine whether it is exclusive of the small vessels or a general vascular pathology that affects all kind of vessels with dominant expression in the small vessels. In addition, our small study lacks the hemodynamic exploration needed to assess the impact of different types of arterial abnormalities in patients. Finally this is a cross-sectional study; a prospective follow-up of the CADASIL patients with ICA or MCA stenosis would have increased the significance of the study.

## Data Availability Statement

The datasets generated for this study are available on request to the corresponding author.

## Ethics Statement

The studies involving human participants were reviewed and approved by Ethics Committee of Beijing Tiantan Hospital. The patients/participants provided their written informed consent to participate in this study. Written informed consent was obtained from the individual(s) for the publication of any potentially identifiable images or data included in this article.

## Author Contributions

Data collection was performed by CZ, WL, SL, SN, XW, and ZZ. CZ, WL, SL, and ZZ: data analysis. CZ: manuscript writing. ZZ: project design, funding, and critical revision. All authors contributed to the article and approved the submitted version.

## Conflict of Interest

The authors declare that the research was conducted in the absence of any commercial or financial relationships that could be construed as a potential conflict of interest.

## References

[1] Cumurciuc R Henry P Gobron C Vicaut E Bousser MG Chabriat H. Electrocardiogram in cerebral autosomal dominant arteriopathy with subcortical infarcts and leukoencephalopathy patients without any clinical evidence of coronary artery disease: a case-control study. Stroke. (2006) 37:1100–2. 10.1161/01.STR.0000209242.68844.20 16514092

[2] Chabriat H Joutel A Dichgans M Tournier-Lasserve E Bousser MG Cadasil. Lancet Neurol. (2009) 8:643–53. 10.1016/S1474-4422(09)70127-9 19539236

[3] Park JH Kim JM Roh JK. Hypoplastic vertebral artery: frequency and associations with ischaemic stroke territory. J Neurol Neurosurg Psychiatry. (2007) 78:954–8. 10.1136/jnnp.2006.105767 17098838 PMC2117863

[4] Gutierrez J Sacco RL Wright CB. Dolichoectasia-an evolving arterial disease. Nat Rev Neurol. (2011) 7:41–50. 10.1038/nrneurol.2010.181 21221115

[5] Fazekas F Chawluk JB Alavi A Hurtig HI Zimmerman RA. MR signal abnormalities at 1.5 T in Alzheimer's dementia and normal aging. AJR Am J Roentgenol. (1987) 149:351–6. 10.2214/ajr.149.2.351 3496763

[6] Ferguson KJ Wardlaw JM Edmond CL Deary IJ Maclullich AM. Intracranial area: a validated method for estimating intracranial volume. J Neuroimaging. (2005) 15:76–8. 10.1111/j.1552-6569.2005.tb00289.x 15574578

[7] Rost NS Cloonan L Kanakis AS Fitzpatrick KM Azzariti DR Clarke V. Determinants of white matter hyperintensity burden in patients with Fabry disease. Neurology. (2016) 86:1880–6. 10.1212/WNL.0000000000002673 27164662 PMC4873685

[8] Charidimou A Boulouis G Haley K Auriel E van Etten ES Fotiadis P. White matter hyperintensity patterns in cerebral amyloid angiopathy and hypertensive arteriopathy. Neurology. (2016) 86:505–11. 10.1212/WNL.0000000000002362 26747886 PMC4753727

[9] Doubal FN MacLullich AM Ferguson KJ Dennis MS Wardlaw JM. Enlarged perivascular spaces on MRI are a feature of cerebral small vessel disease. Stroke. (2010) 41:450–4. 10.1161/STROKEAHA.109.564914 20056930

[10] Maclullich AM Wardlaw JM Ferguson KJ Starr JM Seckl JR Deary IJ. Enlarged perivascular spaces are associated with cognitive function in healthy elderly men. J Neurol Neurosurg Psychiatry. (2004) 75:1519–23. 10.1136/jnnp.2003.030858 15489380 PMC1738797

[11] Charidimou A Jaunmuktane Z Baron JC Burnell M Varlet P Peeters A. White matter perivascular spaces: an MRI marker in pathology-proven cerebral amyloid angiopathy? Neurology. (2014) 82:57–62. 10.1212/01.wnl.0000438225.02729.04 24285616 PMC3873625

[12] Martinez-Ramirez S Pontes-Neto OM Dumas AP Auriel E Halpin A Quimby M. Topography of dilated perivascular spaces in subjects from a memory clinic cohort. Neurology. (2013) 80:1551–6. 10.1212/WNL.0b013e31828f1876 23553482 PMC3662325

[13] Staals J Makin SD Doubal FN Dennis MS Wardlaw JM. Stroke subtype, vascular risk factors, and total MRI brain small-vessel disease burden. Neurology. (2014) 83:1228–34. 10.1212/WNL.0000000000000837 25165388 PMC4180484

[14] Wardlaw JM Smith EE Biessels GJ Cordonnier C Fazekas F Frayne R. Neuroimaging standards for research into small vessel disease and its contribution to ageing and neurodegeneration. Lancet Neurol. (2013) 12:822–38. 10.1016/S1474-4422(13)70124-8 23867200 PMC3714437

[15] Kovac JD Stankovic A Stankovic D Kovac B Saranovic D. Intracranial arterial variations: a comprehensive evaluation using CT angiography. Med Sci Monit. (2014) 20:420–7. 10.12659/MSM.890265 24625840 PMC3962325

[16] Sanders WP Sorek PA Mehta BA. Fenestration of intracranial arteries with special attention to associated aneurysms and other anomalies. AJNR Am J Neuroradiol. (1993) 14:675–80. 8517358 PMC8333398

[17] Bharatha A Aviv RI White J Fox AJ Symons SP. Intracranial arterial fenestrations: frequency on CT angiography and association with other vascular lesions. Surg Radiol Anat. (2008) 30:397–401. 10.1007/s00276-008-0340-7 18350245

[18] Wollschlaeger G Wollschlaeger PB Lucas FV Lopez VF. Experience and result with postmortem cerebral angiography performed as routine procedure of the autopsy. Am J Roentgenol Radium Ther Nucl Med. (1967) 101:68–87. 10.2214/ajr.101.1.68 6037344

[19] Min JH Lee YS. Transcranial Doppler ultrasonographic evaluation of vertebral artery hypoplasia and aplasia. J Neurol Sci. (2007) 260:183–7. 10.1016/j.jns.2007.05.001 17604054

[20] Morimoto K Nagahata M Ono S Miura H Tsushima F Seino H. Incidence of unilateral distal vertebral artery aplasia: evaluation by combining basiparallel anatomic scanning-magnetic resonance imaging (BPAS-MRI) and magnetic resonance angiography. Jpn J Radiol. (2009) 27:151–5. 10.1007/s11604-008-0313-0 19412683

[21] Perlmutter D Rhoton AL Jr Microsurgical anatomy of anterior cerebral anterior communicating recurrent artery complex. Surg Forum. (1976) 27:464–5. 1019940

[22] Zampakis P Panagiotopoulos V Petsas T Kalogeropoulou C. Common and uncommon intracranial arterial anatomic variations in multi-detector computed tomography angiography (MDCTA). What radiologists should be aware of. Insights Imaging. (2015) 6:33–42. 10.1007/s13244-014-0381-x 25680324 PMC4330235

[23] Kang HG Kim JS. Intracranial arterial disease in CADASIL patients. J Neurol Sci. (2015) 359:347–50. 10.1016/j.jns.2015.11.029 26671140

[24] Choi EJ Choi CG Kim JS. Large cerebral artery involvement in CADASIL. Neurology. (2005) 65:1322–4. 10.1212/01.wnl.0000180965.79209.50 16247072

[25] Singhal S Markus HS. Cerebrovascular reactivity and dynamic autoregulation in nondemented patients with CADASIL (cerebral autosomal dominant arteriopathy with subcortical infarcts and leukoencephalopathy). J Neurol. (2005) 252:163–7. 10.1007/s00415-005-0624-3 15729521

[26] Liem MK Lesnik Oberstein SA Haan J Boom R Ferrari MD Buchem MA. Cerebrovascular reactivity is a main determinant of white matter hyperintensity progression in CADASIL. AJNR Am J Neuroradiol. (2009) 30:1244–7. 10.3174/ajnr.A1533 19270103 PMC7051325

[27] Vernooij MW van der Lugt A Ikram MA Wielopolski PA Vrooman HA Hofman A. Total cerebral blood flow and total brain perfusion in the general population: the Rotterdam Scan Study. J Cerebral Blood Flow Metabol. (2008) 28:412–9. 10.1038/sj.jcbfm.9600526 17622253

[28] Fu JH Lu CZ Hong Z Dong Q Ding D Wong KS. Relationship between cerebral vasomotor reactivity and white matter lesions in elderly subjects without large artery occlusive disease. J Neuroimaging. (2006) 16:120–5. 10.1111/j.1552-6569.2006.00030.x 16629733

[29] Bakker SL de Leeuw FE de Groot JC Hofman A Koudstaal PJ Breteler MM. Cerebral vasomotor reactivity and cerebral white matter lesions in the elderly. Neurology. (1999) 52:578–83. 10.1212/WNL.52.3.578 10025791

[30] Miao Q Paloneva T Tuominen S Poyhonen M Tuisku S Viitanen M. Fibrosis and stenosis of the long penetrating cerebral arteries: the cause of the white matter pathology in cerebral autosomal dominant arteriopathy with subcortical infarcts and leukoencephalopathy. Brain Pathol. (2004) 14:358–64. 10.1111/j.1750-3639.2004.tb00078.x 15605982 PMC8095747

